# Ontologies4Cat: investigating the landscape of ontologies for catalysis research data management

**DOI:** 10.1186/s13321-024-00807-2

**Published:** 2024-02-07

**Authors:** Alexander S. Behr, Hendrik Borgelt, Norbert Kockmann

**Affiliations:** Laboratory of Equipment Design, Faculty of Biochemical and Chemical Engineering, TU-Dortmund University, Emil-Figge-Strasse 68, 44139 Dortmund, NRW Germany

**Keywords:** Ontology collection, Research data management, Catalysis, Semantic web, Ontology classification, Metadata

## Abstract

As scientific digitization advances it is imperative ensuring data is Findable, Accessible, Interoperable, and Reusable (FAIR) for machine-processable data. Ontologies play a vital role in enhancing data FAIRness by explicitly representing knowledge in a machine-understandable format. Research data in catalysis research often exhibits complexity and diversity, necessitating a respectively broad collection of ontologies. While ontology portals such as EBI OLS and BioPortal aid in ontology discovery, they lack deep classification, while quality metrics for ontology reusability and domains are absent for the domain of catalysis research. Thus, this work provides an approach for systematic collection of ontology metadata with focus on the catalysis research data value chain. By classifying ontologies by subdomains of catalysis research, the approach is offering efficient comparison across ontologies. Furthermore, a workflow and codebase is presented, facilitating representation of the metadata on GitHub. Finally, a method is presented to automatically map the classes contained in the ontologies of the metadata collection against each other, providing further insights on relatedness of the ontologies listed. The presented methodology is designed for its reusability, enabling its adaptation to other ontology collections or domains of knowledge. The ontology metadata taken up for this work and the code developed and described in this work are available in a GitHub repository at: https://github.com/nfdi4cat/Ontology-Overview-of-NFDI4Cat.

## Introduction

As digitization of the scientific community advances, the need for FAIR (Findable, Accessible, Interoperable, Reusable) [1] data rises to ensure machine-processability of data. Enabling a higher data FAIRness, ontologies represent knowledge explicitly in a machine-understandable way. Ontologies are a collection of machine- and human-interpretable concepts and relations that represent entities and their interdependence in a specific domain [1, 2]. Furthermore, research data occurring in the field of catalysis research often is complex and diverse. Thus, further ontology development and insights for the catalysis research domain are needed [3, 4].

To enhance semantic interoperability and compliance with existing ontologies, a collection of ontologies and semantic artefacts was created with importance to the data value chain of catalysis research. In addition, these ontologies and semantic artefacts were classified regarding their respective subdomains of research shaping the landscape of ontologies for catalysis research [5].

Domain and ontology experts browse and find the proper ontologies for their respective use usually with the help of portals, such as EBI OLS [6] and BioPortal [7]. However, these portals do not provide deep and classification on the ontologies. For example, the knowledge domains represented by an ontology are not covered by any of the two services. This means, that a pre-classification of the ontologies regarding the covered knowledge domain(s) is missing, yet desired. While there are general quantity metrics available, such as, e.g., number of contained classes in an ontology, quality metrics regarding the (re-)usability of ontologies are missing, such as if and which reasoning machine works on the ontology.

In 2022, Strömert et al. [8] screened 22 ontologies representing concepts for research data management in chemistry, also focussing on the (re-)usability, in the context of the NFDI4Chem project. As the authors wants to foster FAIR research data management in chemistry, the publication provides an overview on existing chemistry ontologies, evaluating them against criteria derived from the FAIR principles. Criteria for the evaluation of the ontologies include findability and accessibility of the ontologies, the modularity and alignment to an upper level ontology, as well as the availability of license information. For ontologies to be in scope of the survey, they need to contain a defined set of chemical sub-disciplines, made by domain experts, published and maintained in a FAIR way as well as being used in established applications. Furthermore, advantages and disadvantages of each ontology are discussed and areas of further improvement are highlighted, such as alignment with other ontologies for improved usefulness of the ontologies for FAIR data management [8].

Further classification of ontologies is done in the AIOTI Ontology Landscape Report [9]. Here, a comprehensive and thorough analysis of existing ontologies in the field of Internet of Things (IoT) and Artificial Intelligence (AI) takes place. The report also has a focus of the potential for interoperability and standardization of the ontologies. A total of 31 ontologies are analyzed and an overview of this is given with direct links to more detailed review documents for each ontology. Moreover, ontologies are evaluated on, among others, their functionalities, level of expressivity, and the technology readiness level. However, the report mainly lists ontologies from the domains healthcare, smart cities, energy, agriculture, and transportation. While the methodology and the metadata of the ontologies presented in [9] are well posed and the surveys are conducted thoroughly, most of the ontologies investigated possess minimal or negligible intersection with the domain of catalysis.

Another approach in collecting ontology metadata is realized by the OBO Foundry Dashboard [10], providing insights and metrics of ontologies within the Open Biological and Biomedical Ontologies (OBO) Foundry [11]. The dashboard provides a range of insights and metadata related to the ontologies, such as reuse of the respective ontologies in other ontologies. However, the dashboard only provides ontologies contained within the OBO library and the metrics only focus on (re-)usage related factors. Furthermore, the dashboard does not contain information on the respective scopes of the ontologies with regard to knowledge domains.

The initial listing and the classification of the ontologies and semantic artefacts presented in [5] was not sufficient. In addition, some of these ontologies are not easily reusable and do not provide proper documentation, as also denoted in [8]. As the work presented in [8] provides an overview of ontologies but with regards to the chemical domain of research, the general approach is used as inspiration for this work and is extended accordingly. Additionally, methods regarding the summarizing of ontologies as presented in [9] are considered in this work. Thus, this work presents a reiteration of the initial ontology landscape for catalysis research [5], focusing also on the classification of ontologies and other metadata important for the application of the ontologies listed. While focussing on comprehensibility of the resulting classification of ontologies, the workflow and software is developed to be as reusable as possible, to enable other domains for such ontology classification. Furthermore, a method is developed for a “lightweight” mapping of ontology classes and applied to the investigated ontologies.

**Table 1:** Classification scheme of the ontologies

Section	Content
General information on the ontology	Ontology name; alternative names; ontology acronym; creator(s) and issuing organization; kind of organizational structure
References	Organizational website; persistent URI of ontology file; link to documentation; link to version directory; additional links
Ontology modeling and availability	Provided ontology formats (ttl, owl,...); degree of inference and composition (inferred, non-inferred, compacted,...); license; working reasoners; shortest reasoning time; alignment with top level ontology; ontology imports; prefixes used; class annotation types
Classification of contained domains of interest	Biocatalysis; heterogenous catalysis; homogenous catalysis; photocatalysis; electrocatalysis; chemical substance modeling; material modeling; process modeling; synthesis data; operando data; performance data; characterisation data; heat, transport and kinetic data; process design; energy and cost data; top level ontology
Ontology characteristics	Axioms; logical axiom count; declaration; class count; object property count; data property count; individual count; annotation property count
Comments	Any additional comments or remarks on topics not covered by the other topics

Information regarding the six spreadsheet sections is gathered for each ontology to classify the ontologies regarding the content of each spreadsheet section

## Methods

### Ontology metadata collection for domain relevance of ontologies

To identify suitable ontologies, ontologies listed in the EBI OLS [6] and BioPortal [7] are screened by look-up of classes and keywords by domain and ontology experts. Additionally, the ontologies listed in [5] are considered where suitable together with the overview on the landscape of ontologies in chemistry [8]. The ontology survey is conducted with the help of an intuitively designed spreadsheet template in Microsoft Excel to simplify access and handling of the ontology collection, capturing the relevant information on each ontology. This collection of ontology metadata with focus on the domain relevance is conducted for each ontology. Thus, for each ontology such a template is filled in consisting of six sections listed in Table 1 along the content included in each section.

In the following subchapters, the six spreadsheet sections that were evaluated for the ontologies are elucidated. Furthermore, the respective entries on the content listed in Table 1 are explained.

#### Spreadsheet section: general information on the ontology

To collect general information on the ontology, the ontology name and alternative names are gathered, if they exist. As ontologies often are referred to via their acronym, and stored using the acronym, it is also taken into the metadata. To get insights, whether or not the ontology still is maintained and if the ontology was developed by a consortium or a single person, metadata on the creator(s) and issuing organization is taken into account as well as the kind of organizational structure that developed the ontology.

#### Spreadsheet section: references

The section References of the scheme is intended to collect predominantly Uniform Resource Locators (URLs) for more information on the ontology. This encompasses the website of the organization that issues the ontology to get easy access on eventual updates or new releases of the ontology. Furthermore, the persistent URI of the ontology file is provided, which might be one of the most important metadata collected, as this allows for automated read in and manipulation of the ontology file with, e.g., Python. Some of the established Ontology Lookup services do not use persistent URIs and as such often reference older or deprecated versions, content pages and respective information. Further collected information are the links to the documentation of the ontology as well as to a version directory, if they exist. To account for further web resources on the ontology, such as web links to describing publications and the like, a metadata field for additional links is provided.

#### Spreadsheet section: ontology modeling and availability

Another scope of the metadata collection is to account for the availability and modeling depth of the ontologies. As ontologies can exist in different formats, the first metadata field deals with the available formats of the ontology files, such as Terse RDF Triple Language (TTL) [12] or Web Ontology Language (OWL) [13]. Furthermore, reasoning of the ontologies is an important aspect to obtain explicit knowledge from the otherwise only implicit defined knowledge contained in relationships within the ontology. The degree of inference and composition is collected for information on the availability of already inferred versions of the ontology, and whether compacted versions of the ontology are available. As ontologies are often setup in a modular way consisting of multiple sub-ontologies, a compacted version of an ontology contains all modules merged into a single file with no imports from other ontology files. Especially with focus on the reusability of ontologies, information on the respective license is important. Furthermore, the inference machines (reasoner) used on ontologies differ slightly in their execution, leading into some reasoners not working properly on some ontologies, implying a violation of the implied logic. Thus, the metadata field working reasoners captures the names of the reasoners that work on the ontology and contain empty entries, if there is no reasoner found working on the ontology. Only if the reasoning works for at least one reasoner, the shortest reasoning time can be captured as additional metadata field. Additionally, information about alignment with top level ontologies, such as the Basic Formal Ontology [14], ontologies that are imported via import statements into the ontology are captured by respective metadata fields. The collection of prefixes and class annotation types used in the ontology allows to directly get information on which prefixes are used in the ontology for, e.g., labels of classes.

#### Spreadsheet section: classification of contained domains of interest

As the reuse of an ontology not only depends on its availability and technical circumstances, such as licensing information, a classification with regards to the domains of interest contained within an ontology is also taken into account by the metadata fields. Here, the fields of catalysis research as listed in Table 1 are listed as metadata fields. To decide, whether an ontology enables for classification in the respective subdomain of catalysis research, the entries of the respective metadata fields are filled by screening the class hierarchy of the respective ontology. Where feasible, the textual definitions and annotations of the classes are also considered for the decision on domain relatedness. Furthermore, the available documentation and description of the ontology are taken into account. If many classes contained in the ontology are subject to a subdomain of catalysis research, the entry of the respective subdomain is set to contained, if close to no concepts or no concepts are contained to represent the subdomain, the entry is set to missing. Another classification is done by the rather subjective related:broader and related:narrower concepts, which try to indicate how well the subdomain in question is represented within the ontology.

#### Spreadsheet section: ontology characteristics

Further metadata on the ontology is captured by the section of ontology characteristics, which are aligned with the ontology metrics field of an ontology within Protégé [15]. Here, the number of axioms, logical axiom count, and declaration axioms count provide an idea of the semantic complexity of the ontology. Additionally, the class count, object property count, and data property count are provided to give a more thorough idea on the complexity of the ontology as well as the size. As individuals can provide for examples of the use of classes described within an ontology, the number of individuals also is included. Finally, the annotation property count gives the number of already available annotation properties within the ontology.

#### Spreadsheet section: comments

Any additional comments or remarks on topics not covered by the other topics are gathered within the metadata field of the comments section. This is important, as there might be, e.g., remarks on some of the gathered metadata fields of the other sections, additional information on the metadata collected or additional information about (re-)usability of the ontology in other software.

### Documentation of the recorded metadata

To get the collected metadata of the ontologies into a more representable form whilst also ensuring machine readability of the data, the following workflow is set up. In a first step, the ontology metadata taken up in a Microsoft Excel file, structured as described in Sect. "Ontology metadata collection for domain relevance of ontologies" is converted to Markdown files. Markdown is a lightweight markup language favored for its simplicity and readability and can be rendered automatically on platforms such as GitHub. This provides users with well-formatted and easily accessible content especially used in *readme* files.

It also allows for linking between different Markdown documents, thus interconnecting the metadata aspects. Furthermore, the metadata is converted and stored as JavaScript Object Notation (JSON) files to enable for machine-readability.

The code utilizes the Python Pandas library [16] to ingest the ontology metadata as provided in the Microsoft Excel file. A JSON template is read in that acts as a blueprint for organizing the ontology metadata. The code extracts the information from the Microsoft Excel sheets and integrates it in a Python dictionary setup in the manner of the JSON template. This operation results in a comprehensive representation of each ontology. The resulting JSON data is saved into distinct files, each named after the respective ontology. Markdown files are generated on basis of the Python dictionary, which serve as easy accessible description of each ontology. The main *readme* Markdown file of the repository is also updated. A section within the file is created that contains links to the individual ontology Markdown files. These links and Markdown files are dynamically generated based on the ontologies characterized in the Microsoft Excel file.

Furthermore, the code undertakes an analysis to determine the suitability of ontologies for specific domains of interest of the catalysis research domain as listed in Table 1. This involves classifying ontologies based on their relationships (missing, contained, related:narrower, related:broader) to these domains as described in Sect. "Spreadsheet section: classification of contained domains of interest". To provide visual representations of the ontology relationships, radar plots are generated to categorize ontologies based on the relatedness. This also offers an quick and intuitive way to grasp the connections between ontologies and domains of interest. In addition to radar plots, the code creates Markdown tables summarizing the ontology relationships. These tables are subsequently incorporated into the main *readme* file and accessible to visualize the respective ontologies for each research domain, clustered by the respective relatedness. For each specific ontology, the code produces radar plots tailored to represent its relationships with the domains of interest. Thus, the entire ontology metadata processing workflow is executed, generating the structured documentation of the ontology metadata.

### Ontology mappings

By collecting, among others, not only the relatedness of the ontologies to the respective domains of research, but also the URLs of the ontology raw-files, the collection can be used to automate various tasks regarding ontology analysis. However, it’s worth noting that not all ontologies are provided in the standard OWL syntax, which is the format of ontology files needed for read in using the owlready2 [17] Python package. Thus, an automated conversion takes place where necessary and possible of the ontologies from TTL to the OWL syntax using ROBOT [18]. This ensures that the ontologies are properly loaded into Python with owlready2, allowing for comparison of classes across different ontologies.

The comparison functionality can be viewed as a preliminary mapping of ontology classes, offering an initial assessment of compatibility and relatedness between pairs of ontologies. This provides valuable insights into the potential overlaps and synergies between different knowledge representations. Classes are considered identical if they share the same Internationalized Resource Identifier (IRI), indicating a direct correspondence. Furthermore, classes within ontologies can be named by the annotations name, rdfs:label, rdfs:prefLabel, or skos:altLabel. This presents a challenge in comparing classes, as there can be multiple potential matches not covered by just comparing one way of class annotation against each other. For example, a class might be named via rdfs:label in one ontology, while the same name could be categorized as a rdfs:prefLabel in another. This necessitates a flexible approach to matching ontology classes.

For this, a systematic procedure is followed to streamline ontology comparison by iteration through the ontologies listed in the metadata collection. Using the owlready2 package, a list of classes within the ontologies in Python is retrieved. For each class, compiles a dictionary is compiled that includes the corresponding Internationalized Resource Identifiers (IRIs), along with the associated class attributes name, rdfs:label, rdfs:prefLabel, and skos:altLabel. In cases where one of the attributes is not available, the respective value is filled with none.

This process is repeated for each ontology, and the resulting dictionaries for each ontology are consolidated in an overarching dictionary. This approach eliminates the need to call upon the ontologies each time a comparison is required, accelerating the class comparison in contrast to the alternative of loading each pair of ontologies with owlready2 for every comparison. Then, for each pair of ontologies, the overarched dictionary is called to check for similar entries in the pair of sub-dictionaries for each ontology. To avoid redundancy, matches based on the IRI are searched for first. If a match is found, the class is excluded from further searches for similar name, rdfs:label, rdfs:prefLabel, and skos:altLabel. With this, the method is gathering both the total number of mapped classes and a detailed list of the respective classes from each ontology, facilitating a seamless comparison of the resulting mappings.

While it gives a hint of a potential mapping between classes, the decision only takes place based on same annotation (such as the name of the class). This neglects potential class definition strings or their embedding in the semantic web. Thus, the method presented serves as a “lightweight” approach to mapping ontology classes, offering an initial assessment of their compatibility and interconnection.

Moreover, the code extends its utility beyond automated comparison. Users have the option to apply the same method to their own lists of concepts, enabling the identification of the most suitable domain ontologies for a specific research area related to catalysis research, as defined by the concepts provided by the user. Another notable feature of the method is its capability to utilize the latest versions of ontologies for comparison. This functionality addresses a limitation of existing tools, such as BioPortal, which does not provide automated comparison of the most up-to-date ontology versions at the time of this work’s publication.

**Table 2:** Listing of the ontologies selected for further screening in this work

AFO [19]	CHMO [20]	ISO 15926* [21]	OBI [22]	OSMO [23]
BAO [24]	CIF [25]	ISO 15926-14* [26]	OFM* [27]	PIMS-II* [28, 29]
BFO [14]	DOLCE* [30]	M3 [31]	OM [32]	REX [33]
CAO [34]	EDAM [35]	M4I [36]	OntoCAPE [37]	RXNO [38]
ChEBI [39]	EMMO [40]	MOP [41]	OntoCompChem* [42]	SBO [43]
CHEMINF [44]	ENVO [45]	MS [46]	OntoKin* [47]	VIMMP [48]

Entries with an asterisk(*) denote the onologies not concerned any further for the modelling of catalysis research

## Results and discussion

As described in Sect. "Introduction", the listings and different sources of ontology collections are screened and the metadata of the ontologies are collected using a template as described in Sect. "Ontology Metadata collection for domain relevance of ontologies". With this, a total of 30 ontologies are selected for further screening. They were obtained by search for domain specific keywords using OLS and BioPortal as well as regular web search engines and/or imported ontology classes within the found ontologies. Out of these, a decision was made to exclude seven of these ontologies. This decision was based on issues uncovered during the screening process, primarily related to the availability and accessibility of ontology files. For instance, some of the ISO 15,926 ontology files are proprietary, making it not freely accessible for further metadata collection. Additionally, upon closer examination, some ontologies exhibit lower domain relevance, are outdated and/or no version of the ontology file could be found. Table 2 lists the 30 ontologies, while the seven neglected ontologies are denoted with an asterisk(*) entries resulting in a total of 23 ontologies investigated in the metadata collection.

### Investigating the ontology metadata

The analysis of the metadata of the ontologies reveals several observations regarding their expressivity in the research domains of catalysis research. First, it becomes evident that the domain of catalysis research itself lacks of uniformity, and the existing ontologies fall short in providing thorough descriptions of the research domains. Only four subdomains (Characterisation Data, Chemical Substance Modelling, Material Modelling, and Process Modelling) are described in multiple ontologies. In contrast, most subdomains lack a large number of matching ontologies. The domains Biocatalysis, Operando Data, Performance Data, and Process Design, Energy and Cost Data have only a single ontology that has the concepts of the respective domain contained. In addition, the domains Heterogeneous Catalysis, Homogeneous Catalysis, Photocatalysis, Electrocatalysis, Synthesis Data, and Heat, Transport and Kinetic Data have no dedicated ontology. This makes four of the 14 domains of catalysis research deemed as contained in multiple ontologies, while additional four domains are at least contained in a single ontology, while six domains are not described by any ontology.

**Fig. 1:**
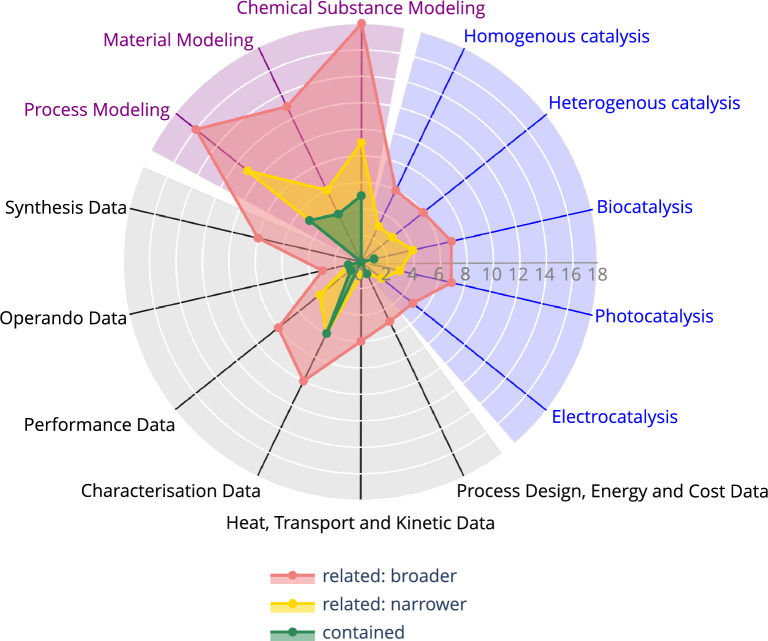
Radar plot for the amount of ontologies that address the respective domains of catalysis research. The specific fields of catalysis are denoted in blue, while the fields more directed to modelling are colored purple. Fields regarding general catalytic data are written in black

Figure 1 shows the number of ontologies related to the respective domain of catalysis research in a radar plot, using the Python Plotly library [49].

**Fig. 2:**
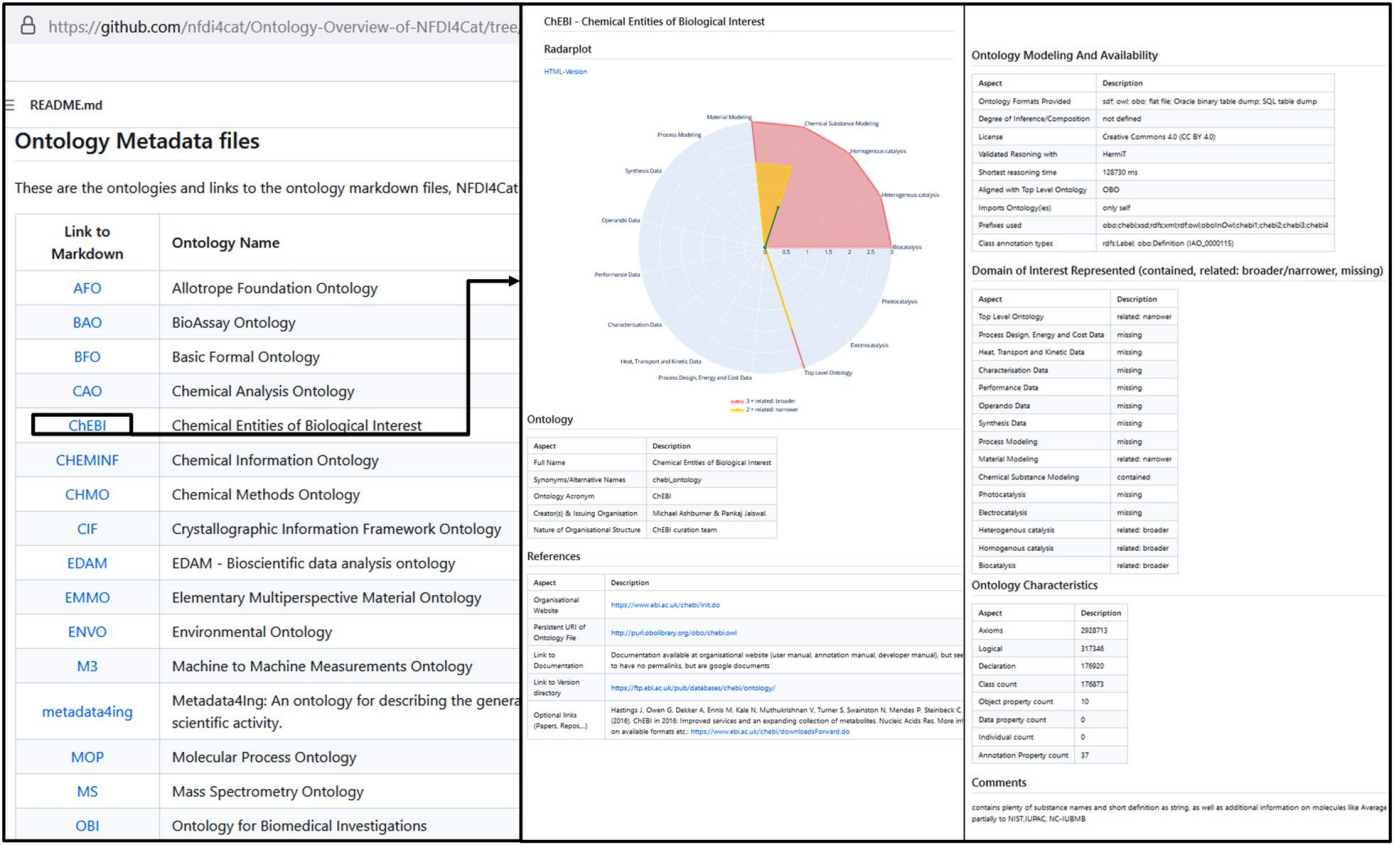
Visualization of the ontology classification via Markdown files on GitHub. The Markdown rendering of the repository *readme* file (left) lists the ontologies and links to the Markdown files describing the respective ontology (right) according to the classifications listed in Table 1 as well as the radar plot, visualizing the respective domains of catalysis research specific to the ontology (top right)

Red denoted are the number of ontologies that are at least related:broader, yellow depicts the number of ontologies that are at least related:narrower, and green depicts the number of ontologies that have the respective domain of catalysis research contained. The specific fields of catalysis are denoted in blue, while the fields more directed to modelling are colored purple. Fields regarding general catalytic data are written in black.

Using multiple ontologies to model a domain of knowledge of catalysis research enables a more nuanced representation of diverse subdomains as more concepts might be contained in the respective ontologies to model the domain. Additionally, it is important to highlight that some of the listed ontologies pose challenges in terms of reasoning, as neither HermiT [50] nor FaCT++ [51] were able to effectively process them. Furthermore, the expressivity of some ontologies should be questioned, as the number of classes diverge widely in the different ontologies. This indicates a need for further refinement and development in this area of ontology engineering to ensure robust and comprehensive coverage within the field of catalysis sciences.

To facilitate documentation and ease access, the content of the ontology metadata listed in the Microsoft Excel file is used to automatically generate Markdown files that contain simplified, text-based formatting instructions and can be rendered similarly to Hypertext Markup Language (HTML). Rendering the Markdown files in GitHub provides a comprehensive and interactive overview of each ontology, making it easier for researchers to assess the suitability of an ontology for their research needs. Thus, the structure of the repository [52] is outlined below. The landing page of the repository shows the main *readme* file, which is formatted in Markdown syntax. It provides an overview on the whole ontology metadata collection, such as the radar plot shown in Fig. 1. Furthermore, a listing of the 23 ontologies is provided, containing the abbreviation and the full name of the ontologies. As Markdown allows for interlinking of files, the abbreviations of the ontologies link to separate Markdown files. Beside the general metadata of the ontology, a radar plot is contained in these Markdown files, showing the categorization of the respective ontology into the 14 domains of catalysis research similar to the one presented in Fig. 1. An excerpt of the rendering of the main *readme* Markdown-file and excerpts of the rendered page for the ChEBI ontology are depicted exemplarily in Fig. 2.

The data is also exported via Python to JSON files, by assigning key-value pairs of the respective metadata fields. This increases the machine-readability of the results, which eases further use of the data, such that other software can easily read out the metadata of the ontologies. Finally, the main *readme* Markdown-file also contains an overview on the mappings generated for pairs of ontologies. This overview and the respective results are described in the following section.

### Mapping of ontology classes

**Fig. 3:**
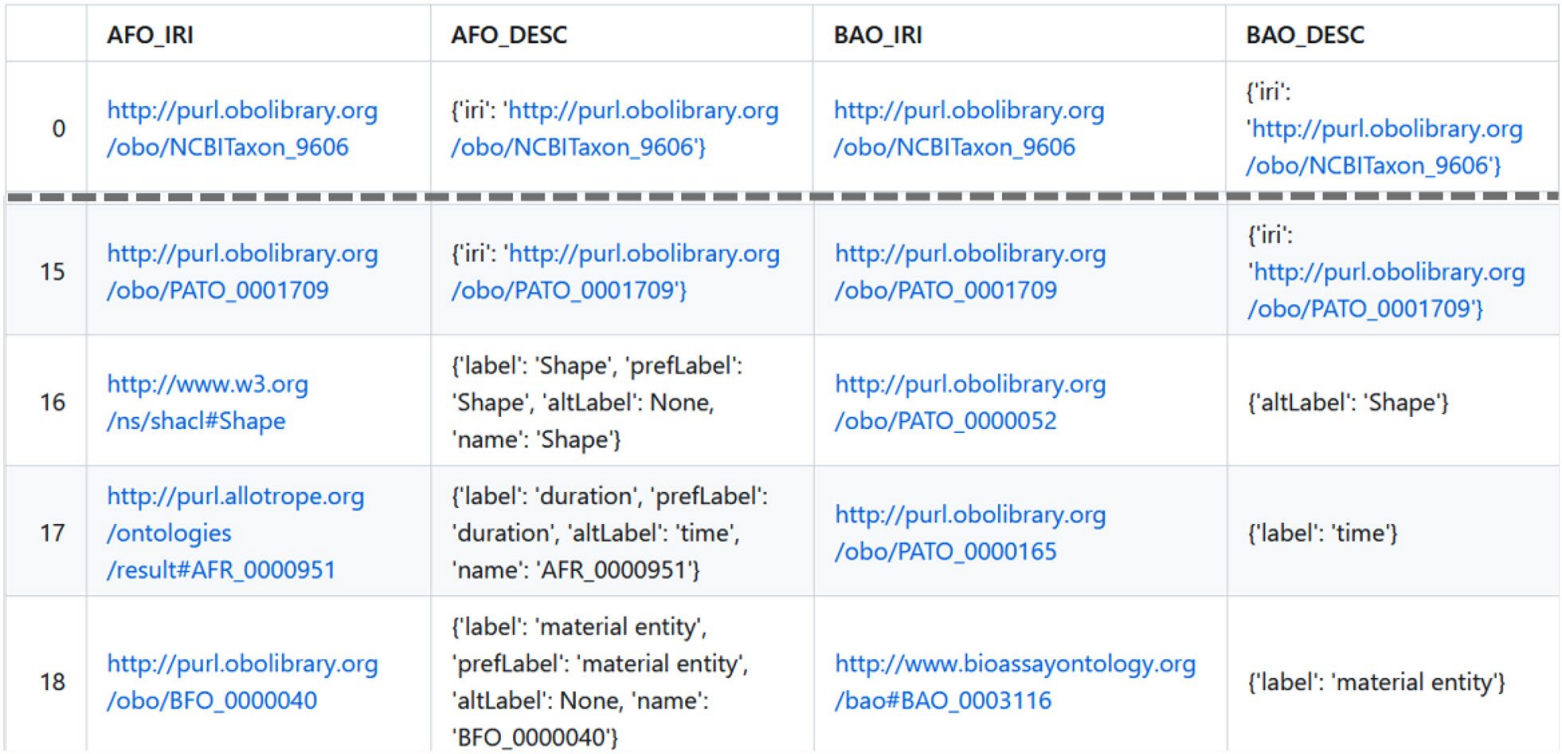
Exemplary excerpt of mapping of AFO and BAO ontologies, converted as Markdown file and rendered via GitHub. The grey dashed line denotes a jump in the list, as the first 16 entries (entry 0–15) show mappings because of same class IRIs, while the following entries show mappings due to same annotations of classes

The mapping of classes between ontology pairs is performed for the investigated ontologies except for OntoCAPE. This is due to the modular and deprecated design of the ontology, which made it impossible for the authors to load it properly with owlready2. Thus, a total of 22 ontologies are subject to the mapping described in Sect. "Ontology mappings". Table 3 lists the resulting number of classes mapped for each pair of ontologies. The main diagonal of the table lists the total amount of classes for each ontology. Exemplarly, the method found 107 similar classes (entry underlined in Table 3) in the Allotrope Foundation Ontology [19] (AFO, total of 2876 classes) and BioAssay Ontology [24] (BAO, total of 7512 classes) ontologies. The table of mapped classes is also included in the main *readme* file of the repository, providing an interactive interface via Markdown syntax.

**Table 3:** Resulting number of classes mapped for each pair of ontologies

	AFO	BAO	BFO	CAO	ChEBI	CHEMINF	CHMO	CIF	EDAM	EMMO	ENVO	M3	M4I	MOP	MS	OBI	OM	OSMO	REX	RXNO	SBO	VIMMP
AFO	2876																					
BAO	107	7512																				
BFO	36	4	35																			
CAO	121	25	14	445																		
ChEBI	58	1678	1	45	176873																	
CHEMINF	92	14	35	42	2	850																
CHMO	249	39	12	69	23	19	3101															
CIF	128	24	4	19	12	37	6	32														
EDAM	50	35	0	12	3	19	9	15	3473													
EMMO	144	21	4	22	23	36	10	502	14	935												
ENVO	248	212	26	84	939	63	36	62	21	64	6566											
M3	88	27	0	19	9	8	6	67	2	65	389	761										
M4I	18	2	3	7	1	4	3	7	1	13	5	10	32									
MOP	6	7	3	8	58	3	3	3	0	3	25	0	1	3686								
MS	140	47	0	26	20	28	30	35	26	31	75	61	1	1	14989							
OBI	289	172	35	82	136	236	77	61	48	54	399	97	6	6	55	4866						
OM	100	21	1	17	11	21	2	80	5	78	226	131	4	0	24	81	815					
OSMO	8	1	0	2	0	8	0	5	4	4	13	4	1	0	3	19	5	173				
REX	9	7	0	2	0	0	18	0	0	1	16	6	1	23	2	7	1	0	552			
RXNO	14	8	2	17	230	5	10	5	0	4	95	1	1	123	3	16	0	0	12	1019		
SBO	41	27	2	7	13	9	3	14	7	17	62	11	1	20	9	31	31	1	11	8	694	
VIMMP	83	13	3	19	3	33	5	83	15	90	74	37	8	1	12	96	72	172	0	2	9	1082

The main diagonal of the table lists the total number of classes contained in the respective ontology. Exemplary for the pair of AFO and BAO ontologies, 107 similar classes were found (underlined in table). An interactive version of this table via Markdown can be found in the main *readme* of the GitHub repository [52]

There, numbers within the table are clickable, redirecting users to dedicated Markdown files. These files in turn list the detailed information on the mappings between respective ontology pairs in a table. The first two columns record the class IRI and rationale for the mapping (IRI or associated class attributes) from the first ontology. Similarly, the next two columns document the same data taken from the mapped class of the second ontology. Finally, the last column contains the textual definition of the class of the second ontology, where available. This ensures a comprehensive and transparent documentation of the mapping process, allowing for easy access and review of mapped classes. Figure 3 shows an exemplary excerpt of such a Markdown file containing details of the mapping between the two ontologies AFO [19] and BAO [24]. The overall number of mapped classes between those two ontologies is 107 as listed in Table 3. For simplicity, only five classes are presented here, showing the structure of the resulting mapping tables. First, classes are listed that are mapped based on the same IRI. These classes have the same IRI and thus are mapped accordingly. In this example, from entry 16 on, the mappings based on the associated class attributes are listed. The class with the rdfs:label *Shape* in the AFO is mapped to a class in the BAO with a skos:altLabel entry *Shape*. Furthermore, the next listed class with the skos:altLabel *time* in the AFO is mapped to a class in the BAO with a rdfs:label entry *time*. By clicking on the IRI, users are can get deeper insights on the ontology class as hosted by the ontology providers.

## Conclusion

This work presents a workflow to setup metadata of ontologies with focus on the domain relevance and display current data for comparison. The metadata is recorded with regards to specific domains of knowledge that extends the data usually presented in ontology databases such as EBI OLS [6] and BioPortal [7]. Furthermore, a codebase is presented that transfers the collected metadata automatically into easy to read Markdown files. Integration into GitHub facilitates visual representation of the metadata and provides quick insight into those ontologies that are most relevant to a particular knowledge domain. The metadata and comparison is made accessible through a GitHub repository and also exported as JSON files for machine-readability. The overall systematic method offers efficient means of comparison across ontologies from a domain of knowledge. Thus, the implemented code and metadata templates aim to be as reusable as possible, to allow for further adaptation on other domains of knowledge.

By dividing into 14 subdomains in three areas, this way of collecting ontology metadata is shown for the domain of catalysis research. With this, a total of 30 ontologies were selected for further screening, but seven were excluded due to accessibility issues or lack of relevance to the domain. The remaining 23 were investigated revealing varying levels of complexity and coverage across different domains within catalysis research. The classification included, among others, the relatedness of the ontologies to each of the 14 subdomains. Relatedness to each subdomain was ranked by four categories; contained, related:narrower, related:broader, and missing. This revealed a graphic representation of the ontologies’ metadata for catalysis research, as depicted in Fig. 1. While four subdomains were connected to multiple ontologies, ten were only modeled by one or none. Furthermore, some ontologies posed challenges in reasoning and have differing levels of expressivity. This emphasizes the need for more ontologies or more extended ontologies to describe the domain of catalysis research in more detail.

An approach for automated mapping of classes between ontologies is described, showing potential mappings between classes of overall 22 ontologies related to catalysis research. The results of the mapping are also represented in GitHub for better accessibility and readability in Markdown files. Moreover, Markdown files are created for each pair of ontologies, listing the classes and reasons for mapping of the classes of both mapped ontologies for further review.

While searching for similar class annotations might give a hint on possible class mappings between two ontologies, a user-controlled revision of these mappings should take place. As this task is quite tedious, automation of this process with other code-based solutions should be investigated further. For example, a comparison of the textual, often sentence-wise definitions of a class could be taken into account. A promising technique is described by Korel et al. [53] which could be used in future work to help in automated mappings of ontology classes by similar textual definitions. However, this will only help in mappings of classes, where those definitions are provided, which is often not the case. Here, mapping techniques could be applied, that also considers the interconnection of the class candidates in their respective ontologies.

## Data Availability

The files contained in the following GitHub directory are available free of charge. The ontology metadata taken up for this work and the code developed and described in this work are available in a GitHub repository at: https://github.com/nfdi4cat/Ontology-Overview-of-NFDI4Cat The metadata collection might be subject to change due to further contributions. Thus, the state of the repository as described in this work is available on Zenodo https://zenodo.org/doi/10.5281/zenodo.10470987 convert.py: contains the Python methods described in Sect. "Documentation of the recorded metadata" to convert the ontology metadata to the respective Markdown files and generate the radar plots. similarities.py: contains the Python methods described in Sect. "Ontology mappings" to conduct the automated mapping of classes of different ontologies. The repositories subdirectory ./master_table/ contains the Microsoft Excel file to take up the ontology metadata.

## Declarations

A part of this work with preliminary and deprecated results has been published as conference talk and its abstract [54].
